# Research Directions for Leveraging and Supporting the Lived Experience of Mental Illness within Psychology

**DOI:** 10.3390/healthcare11162318

**Published:** 2023-08-17

**Authors:** Darren Haywood, Frank D. Baughman, Peter Bosanac, Kim Johnston, Inge Gnatt, Jennifer Haywood, Judith Gullifer, Susan Rossell

**Affiliations:** 1 School of Psychological Sciences, Turner Institute of Brain and Mental Health, Monash University, Clayton, VIC 3800, Australia; 2 Department of Mental Health, St. Vincent’s Hospital Melbourne, Fitzroy, VIC 3065, Australia; peter.bosanac@svha.org.au (P.B.);; 3 Department of Psychiatry, University of Melbourne, Parkville, VIC 3052, Australia; 4 School of Population Health, Curtin University Western Australia, Bentley, WA 6102, Australia; 5 Centre for Mental Health and Brain Sciences, Swinburne University of Technology, Hawthorn, VIC 3122, Australia

**Keywords:** lived experience, mental health, psychology, education, university, students, clinicians, peer workers

## Abstract

This paper explores the lived experience of mental illness within the field of psychology across higher education and the mental health workforce. There is a high prevalence of mental health issues among psychology students and practitioners, and it is critical not only to provide support for these populations, but also to acknowledge the value of leveraging their lived experience within their education and practice. There has been increased interest in and advocacy for the involvement of those with lived experience of mental illness within mental healthcare service provision to improve patient experiences and outcomes. However, there have been limited acknowledgement and research regarding the role of psychologists with personal lived experiences of mental illness, and how to leverage this experience. Further, there are challenges faced by both psychology students and practising psychologists with lived experience that act as barriers to leveraging their unique skills and experiences. Psychology students with lived experience face stigma, inadequate support, and incongruence between the course material and their personal experiences. Similarly, practising psychologists with lived experience encounter stigma and isolation, indicating the need for a culture change that promotes transparency and understanding. The paper calls for research in five key directions to provide evidence that can be used to support and leverage lived experience in psychology.

## 1. Introduction

In recent years, there has been increased interest in and acknowledgement of the lived experience of mental illness in future (i.e., student/trainee) and current health professionals. Lived experience, which includes having personal experience in both the past and/or present (i.e., living experience) of mental ill-health (diagnosed or otherwise), within mental health specialities such as psychology is particularly interesting given its direct relevance to service provision. Within psychology student populations, there has been an acknowledgement of the high prevalence of poor mental health and an emerging focus on providing systems of support. Likewise, in mental health service provision, there has been a focus on the utility of lived experience workers (i.e., peer workers without clinical qualifications) in clinical practice. However, to date, there is limited acknowledgement and focus on the potential utility of the lived experience of mental illness for future and current psychologists. Developing best practices to support these populations throughout their career journey from student to clinician, while leveraging their unique skillsets and insight, has the potential to facilitate better educational and clinical outcomes and boost career longevity. Given the resource limitations in mental healthcare worldwide, leveraging and supporting existing skillsets and experiences within the mental health workforce is of paramount importance. In this paper, we the authors, who are members of a variety of key stakeholders, including clinical mental health service directors, directors of education in psychology, practising psychologists, lecturers in psychology, undergraduates, and psychological scientists, provide our perspective on the literature in this area and directions for research progression. Specifically, we explore the lived experience of mental illness within the field of psychology across higher education and the mental health workforce and provide five key research directions that, given the current paucity of the directed research in this area, may provide evidence that can be used to support and leverage the lived experience of mental illness within the discipline of psychology.

## 2. Background

Within higher education, poor mental health is common [1,2]. For example, over one-quarter of undergraduates have depression at any given time [1,2] and the peak onset of mental illness and initial diagnoses is found in the age group typically undertaking higher education (i.e., 18–25 years of age; [3]). In recent years, exacerbated by the COVID-19 pandemic, poor mental health, including the prevalence of disorders and the severity of mental health symptoms, within higher education settings continues to be commonplace [1,4,5]. Students frequently choose to undertake a university course based on their personal interests, often influenced by previous life experiences, e.g., [6]. In line with this, students undertaking university studies in psychology regularly report having lived experience of mental illness and choose to pursue study in this field to improve their understanding of their experience and aspire to help others who also experience mental illness [7,8]. Consequently, across the psychology discipline, from students to practitioners, lived experience of mental illness is common. For instance, Victor et al. [9] found that over 82% of higher education psychology faculty and students (e.g., graduate students, school faculty, clinicians, and post-doctoral students) across the United States and Canada reported experiencing mental health difficulties, and almost half of the sample reported having been formally diagnosed with a mental disorder. Similarly, Tay et al. [10] found that two thirds of clinical psychologists reported personal experiences with mental health problems. Still, despite evidence suggesting a strong presence of lived experience within student cohorts, robust ethical boundaries existing within psychological practice (i.e., where staff may be hesitant to ask about mental health problems to ensure dual relationships are avoided) and other faculty pressures (e.g., increased work demands or fiscal policy) may prevent adequate support from being provided within higher education. Beyond higher education, psychologists working in public and private healthcare settings may report similar levels of lived experience, however, this is not yet clear. Reasons for this lack of clarity may be associated with the limited research investigating this topic, and also related to the perception that those who do disclose may encounter negative consequences and additional challenges. These include discrimination and reduced opportunities in the workforce (i.e., not being hired due to potential under performance or absence due to mental illness) [11]. Due to stringent rules regarding impairment that may occur alongside the experience of a mental illness, psychologists could also risk their professional license/registration. Shame and perceived mental health stigma from others have also been reported as barriers to disclosing and help seeking [10]. Moving forward, it is crucial to support psychology students’ and practitioners’ mental health, and important to recognise the lived experiences of psychology students and practitioners with mental illness as a strength. Supporting students to encompass their lived experience into their professional careers has the potential to nourish the calibre, diversity, and longevity of the future workforce.

To date, the presence of lived experience has been acknowledged in some training and professional settings, for example, through consultations in decision making and support through supervision. However, there is a paucity of data relating to the implementation and subsequent assessment of the utility of including lived experiences in psychological strategies or treatment programmes. Recently, Victor et al. [12] offered a range of practical recommendations aimed at facilitating improved policy and institutional, structural, and practical factors relating to those with lived experience in psychology, for both students and practitioners. Whilst these recommendations have the potential to create an environment in which individuals can thrive at various levels, to date, there has not been an opportunity to deliver and assess their utility. Consequentially, the ability to make evidence-based educational or practice improvements in these areas is currently limited, highlighting the paucity of research and research directions regarding the lived experience of mental illness within the discipline of psychology. To facilitate the required reform, coordinated research is required to better understand the experiences, barriers, and facilitators regarding education and practice for those with lived experience of mental illness in psychology.

In this paper, we discuss key considerations relating to the inclusion of lived experiences of mental illness within the higher education/university setting and mental health workforce settings, specific to psychology, and conclude by providing five key directions for future research. We argue that an increased understanding within these five directions of inquiry is required to (a) properly support students, trainees, and professional and research psychologists in the future, and (b) successfully leverage/integrate the unique skills and experiences provided by those with lived experiences of mental illness within psychology.

## 3. Lived Experience

In recent years, there has been increased interest in and advocacy for the involvement of those with lived experiences of mental illness within mental healthcare service provision [13]. For example, recently, in Australia, the Royal Commission into Victoria’s Mental Health System [14] described lived experience personnel, via mechanisms of consultation and co-design, as being central to reforming the mental health workforce and improving patient experiences and outcomes. Typically, lived experience in mental health service provision is provided by ‘peer workers’, non-clinical paid staff, or volunteers with a self-reported lived experience of mental ill-health, e.g., [15]. Lived experience peer workers primarily deliver service to consumers by offering experiential knowledge, helping them to better navigate the health system, providing reassurance and enhancing comfort, and collaborating with clinical staff to optimise patient care [16]. Peer workers have been shown to provide hope, interpersonal connection, experiential validation, de-escalation, and facilitate recovery-oriented practice, as well as improve consumers’ clinical outcomes [16,17,18,19]. Lived experience peer workers may also be engaged in and consulted with for leadership and decision-making positions, where their skills and expertise are integrated into policy and process recommendations, for example, in the recruitment of new staff or developing procedures and processes. To date, peer workers have been utilised in a wide variety of mental health services, including emergency, general clinical psychology, acute in-patient services, and condition-specific services (i.e., eating disorder services; [16,17,19,20]). Similar to peer workers, clinical staff with lived experience of mental illness, such as psychologists, have also described their lived experience as being foundational to building and establishing a close and consistent therapeutic relationship with patients, e.g., [21]. A key difference however, is that the majority of clinical staff, including psychologists, do not feel supported to openly disclose their personal experience of mental illness, which can be isolating [9,22].

## 4. Lived Experience within the Discipline of Psychology

Given the benefit offered by those with lived experience of mental illness within mental healthcare, it is important to acknowledge the potential utility of lived experiences of mental illness within clinical mental health staff and researchers, such as psychologists, and support their education and ongoing practice. It is equally important to acknowledge and reduce the barriers and challenges within education and practice for people with lived experiences of mental illness within the discipline of psychology. For instance, students who experience mental illness may be at a disadvantage to progress, and therefore be excluded from advancing towards future training programs given the competitive nature of entry, which includes high grades and previous experience (e.g., volunteering) and anecdotally excludes individuals who openly disclose [23]. Still, considering the higher proportion of psychology students and staff with lived experience [12], there is an opportunity to work towards providing appropriate methods for leveraging these lived experiences for the benefit of the individual, and ultimately their clients, patients, or consumers. Therefore, it is important for psychology students and staff alike to understand how to leverage their lived experience within their training and service provision, and perceive their lived experience of mental illness as an aid in developing their psychological literacies and, for some, as potentially strengthening their clinical service provision. However, there are currently a number of barriers to disclosure and therefore, support that exist within the training and clinical practice of psychology, for both students and staff with lived experience of mental illness.

### 4.1. Psychology Students with Lived Experience

University students with mental illness face significant and wide-ranging challenges within their education. For example, university students with mental illness experience increased stigma [24], questions regarding the validity and severity of their condition [25], difficulties graduating [23], and poorer-quality relationships with other students and university staff when compared to those without mental illness [26]. There is limited research regarding the prevalence of mental illness among psychology students and their experiences of studying psychology. However, like university students broadly, there also seems to be stigma, a negative attribute that causes personal devaluation, towards psychology students with mental illness. For example, Victor et al. [12] illustrated that ratings of ‘emotional instability’ from non-validated scales are included in many current psychology graduate school application systems and, previously, Appleby and Appleby [27] described the disclosure of mental illness in students’ psychology graduate school applications as a ‘kiss of death’. Woof et al. [8] also found that psychology undergraduate students with lived experience of mental illness described that the delivery of the course content relating to mental disorders often triggered their own mental health condition. The reasons for this could include the othering manner in which the information was provided (i.e., judgemental and stigmatising), a recognition of their own difficulties that had not yet been identified or attended to, or perhaps the triggering of traumatic aspects of their own experiences through the content delivery. Similarly, psychology undergraduate students with lived experience of mental illness have expressed an incongruence between the course material and their experiential knowledge, whereby the course descriptions of mental health conditions did not adequately reflect their lived experience [8]. Woof et al. [8] also found that psychology students experienced challenges obtaining support from teaching staff and the university mental health support services due to factors such as a high staff workload and fellow psychology students working in student support, minimising anonymity. The exploratory research of Woof et al. [8] suggests that, in addition to the typical challenges faced by university students with mental illness (see [23,24,25,26]), psychology students with mental illness encounter additional complexities in engaging and navigating their educational landscape, due to their course content and activities, likely exacerbated by fear of disclosure and the potential impact on their future opportunities.

### 4.2. Psychologists with Lived Experience

Like psychology students with lived experience of mental illness, there is limited empirical research exploring psychologists with lived experience of mental illness, making it difficult to draw firm conclusions in relation to their prevalence and aspects of the working environment [12]. However, like research with peer workers, the limited available evidence suggests that mental health practitioners, including psychologists, with lived experience of mental illness also find their lived experience to be highly useful in their clinical practice and foundational to building and establishing therapeutic relationships with consumers, e.g., [21]. However, research also shows that, once in the workplace, mental health professionals with lived experience of mental illness often continue to face challenges that impact their well-being and the services provided to consumers [28,29,30]. For example, more than three quarters of mental health professionals with lived experience of mental illness surveyed in the United States reported experiencing workplace bullying [29]. Most of these victims of bullying reported that the perpetrator(s) did not know they had lived experience, which could suggest that increased transparency relating to lived experience may be protective by enhancing understanding [29]. However, mental health professionals with lived experience of mental illness face wide-ranging stigma in the workplace from other health professionals, including fellow mental health professionals, further highlighting the need for systemic change [12,22,28,29]. It is suggested that a culture of nondisclosure within healthcare services may contribute to the perceived stigma from others as opposed to external (i.e., towards others) and self-stigma, and isolation faced by those with lived experience [10,28,30]. Although research suggests that the disclosure of the lived experience of mental health professionals may reduce workplace stigma and bullying and deepen connection and understanding, mental health workers with lived experience express concern regarding potential exclusion and discrimination [21]. Psychologists with lived experience working within research settings also warrant attention. The likelihood of these individuals having lived experience is equal to that of students and professionals in clinical settings, yet potentially even more limited in terms of self-disclosure and support. In combination, the body of research suggests that workplace culture change, creating an open, supportive, transparent, and non-judgmental environment in mental health services and research settings regarding lived experience is integral to optimising mental health professional and patient wellbeing.

## 5. Five Key Research Directions

Below, we provide five directions of research that should be pursued to facilitate educational and practice reform to support and leverage the lived experience of mental illness within psychology.

1. **Explore the prevalence of mental illness among psychology students and psychologists across various settings.**

This will provide a more comprehensive view, including the prevalence and levels of disclosure. We recommend this direction of research not be limited to diagnostic status, but should take a dimensional approach to exploring the severity of mental health differences across a range of higher- and lower-level domains of psychopathology [31,32,33,34], as well as potential protective and resilience factors. We suggest that education and healthcare providers are consulted with in the design and recruitment of this portion of the research. Although this will involve a further step and extend the duration, in order to achieve the outcome of increasing inclusivity, all stakeholders should be given the opportunity to participate throughout. Furthermore, it may begin to alleviate some of the perceptions that the stigma towards individuals with lived experience is most prominent from organisations.

2. **Explore the perspectives and experiences of psychology students and psychologists with mental illness.**

This research should adopt qualitative and quantitative approaches to better understand the outlooks and experiences of psychology students and psychologists with lived experiences of mental illness within their educational or workplace settings. This research may explore the barriers to and facilitators for education and practice, as well as experiences of stigma and other psychosocial considerations. This research can adopt exploratory, descriptive, and/or explanatory approaches, including phenomenological, thematic, and predictive studies. Again, we would recommend that all stakeholders be invited to participate in this aspect of the research to further strengthen acknowledgement and participation.

3. **Develop and assess approaches to support psychology students with lived experience of mental illness, and facilitate the utility of their life experiences in their education and future careers.**

This research may explore approaches to reducing stigma, enhancing educational supportive practices, and developing teaching approaches and content, in order to acknowledge and leverage the lived experiences of mental illness within psychology student cohorts. The inclusion of education and healthcare organisations in this process may be useful. For example, there may be opportunities to leverage the knowledge from previous programmes of work that have sought to increase inclusivity (e.g., LGBTQIA+, Indigenous/cultural inclusivity). Furthermore, there may be opportunities for individuals working within these organisations to provide suggestions in terms of what may have been helpful in their own experiences of navigating these systems.

4. **Develop and assess interventions to reduce stigma, bullying, and the culture of nondisclosure of mental illness within mental health services.**

Research in this domain may develop and assess purpose-built interventions, informed by the experiences and perspectives of those with lived experiences of mental illness, aimed at reforming the mental health service culture and approaches to facilitating the potential disclosure of lived experiences of mental illness. These interventions should be informed by previous efforts made in other domains (e.g., LGBTQIA+, Indigenous/cultural inclusivity), which will also inform aspects such as the requirements for funding that will support the success of programmes.

5. **Further explore and acknowledge the utility of psychologists with lived experience of mental illness within the workplace.**

This research may provide an increased understanding of the clinical and research applications and utility of the lived experience of mental illness among psychologists, with the goal of leveraging this experience throughout their employment journey to improve clinical practice and consumer outcomes, and to inform educational practice. From a practical perspective, this could include the development and delivery to inform organisations of the potential benefits of the greater inclusion and support of lived experience, and we suggest that guidelines are produced to assist with this.

## 6. Conclusions

Ultimately, psychology students, researchers, and clinical staff with lived experiences of mental illness offer a unique skill set that may enhance future policy, research, patient care, and ultimately outcomes. To date, there has been limited directed research conducted into how lived experience could be embedded more beneficially across educational, research, and clinical settings, however, it is essential that this is prioritised. Firstly, improved acknowledgement and understanding, systems, and educational approaches are required to effectively support and encourage the progression of students with lived experience studying psychology at university, in order to build a more diverse future workforce. Further, changes in workplace culture aimed at facilitating the potential of lived experience disclosure for psychologists in clinical and research settings, and the subsequent use of their experiential knowledge, are required. We presented five key directions for future research that will facilitate evidence-informed practice aimed at supporting and leveraging the lived experience of mental illness within psychology.

## Data Availability

Not applicable.

## References

[1] Dessauvagie A.S., Dang H.-M., Nguyen T.A.T., Groen G. (2022). Mental health of university students in southeastern asia: A systematic review. Asia Pac. J. Public Health.

[2] Ibrahim A.K., Kelly S.J., Adams C.E., Glazebrook C. (2013). A systematic review of studies of depression prevalence in university students. J. Psychiatr. Res..

[3] Solmi M., Radua J., Olivola M., Croce E., Soardo L., Salazar de Pablo G., Il Shin J., Kirkbride J.B., Jones P., Kim J.H. (2022). Age at onset of mental disorders worldwide: Large-scale meta-analysis of 192 epidemiological studies. Mol. Psychiatry.

[4] Chen T., Lucock M. (2022). The mental health of university students during the COVID-19 pandemic: An online survey in the UK. PLoS ONE.

[5] Jiang J., Akhlaghi H., Haywood D., Morrissey B., Parnis S. (2022). Mental health consequences of COVID-19 suppression strategies in Victoria, Australia: A narrative review. J. Int. Med. Res..

[6] Pagnin D., De Queiroz V., Oliveira Filho M.A.D., Gonzalez N.V.A., Salgado A.E.T., Oliveira B.C.E., Lodi C.S., Melo R.M.D.S. (2013). Burnout and career choice motivation in medical students. Med. Teach..

[7] Marrs H., Barb M.R., Ruggiero J.C. (2007). Self-Reported Influences on Psychology Major Choice and Personality. Individ. Differ. Res..

[8] Woof V.G., Hames C., Speer S., Cohen D.L. (2021). A qualitative exploration of the unique barriers, challenges and experiences encountered by undergraduate psychology students with mental health problems. Stud. High. Educ..

[9] Victor S.E., Devendorf A.R., Lewis S.P., Rottenberg J., Muehlenkamp J.J., Stage D.R.L., Miller R.H. (2022). Only human: Mental-health difficulties among clinical, counseling, and school psychology faculty and trainees. Perspect. Psychol. Sci..

[10] Tay S., Alcock K., Scior K. (2018). Mental health problems among clinical psychologists: Stigma and its impact on disclosure and help-seeking. J. Clin. Psychol..

[11] Brohan E., Henderson C., Wheat K., Malcolm E., Clement S., Barley E.A., Slade M., Thornicroft G. (2012). Systematic review of beliefs, behaviours and influencing factors associated with disclosure of a mental health problem in the workplace. BMC Psychiatry.

[12] Victor S.E., Schleider J.L., Ammerman B.A., Bradford D.E., Devendorf A.R., Gruber J., Gunaydin L.A., Hallion L.S., Kaufman E.A., Lewis S.P. (2022). Leveraging the strengths of psychologists with lived experience of psychopathology. Perspect. Psychol. Sci..

[13] Mutschler C., Bellamy C., Davidson L., Lichtenstein S., Kidd S. (2022). Implementation of peer support in mental health services: A systematic review of the literature. Psychol. Serv..

[14] State of Victoria, Royal Commission into Victoria’s Mental Health System (2021). Final Report.

[15] Hurley J., Cashin A., Mills J., Hutchinson M., Kozlowski D., Graham I. (2018). Qualitative study of peer workers within the ‘Partners in Recovery’ programme in regional Australia. Int. J. Ment. Health Nurs..

[16] Barr K.R., Townsend M.L., Grenyer B.F.S. (2020). Using peer workers with lived experience to support the treatment of borderline personality disorder: A qualitative study of consumer, carer and clinician perspectives. Borderline Personal. Disord. Emot. Dysregulation.

[17] Brasier C., Roennfeldt H., Hamilton B., Martel A., Hill N., Stratford A., Buchanan-Hagen S., Byrne L., Castle D., Cocks N. (2022). Peer support work for people experiencing mental distress attending the emergency department: Exploring the potential. Emerg. Med. Australas..

[18] Chisholm J., Petrakis M. (2022). Peer Worker Perspectives on Barriers and Facilitators: Implementation of Recovery-Oriented Practice in a Public Mental Health Service. J. Evid.-Based Soc. Work.

[19] Solomon P. (2004). Peer support/peer provided services underlying processes, benefits, and critical ingredients. Psychiatr. Rehabil. J..

[20] Beveridge J., Phillipou A., Jenkins Z., Newton R., Brennan L., Hanly F., Torrens-Witherow B., Warren N., Edwards K., Castle D. (2019). Peer mentoring for eating disorders: Results from the evaluation of a pilot program. J. Eat. Disord..

[21] Marino C., Child B., Campbell Krasinski V. (2016). Sharing Experience Learned Firsthand (SELF): Self-disclosure of lived experience in mental health services and supports. Psychiatr. Rehabil. J..

[22] Victor S.E., Lewis S.P., Muehlenkamp J.J. (2022). Psychologists with lived experience of non-suicidal self-injury: Priorities, obstacles, and recommendations for inclusion. Psychol. Serv..

[23] Ishii T., Tachikawa H., Shiratori Y., Hori T., Aiba M., Kuga K., Arai T. (2018). What kinds of factors affect the academic outcomes of university students with mental disorders? A retrospective study based on medical records. Asian J. Psychiatry.

[24] Turosak A., Siwierka J. (2021). Mental health and stigma on campus: Insights from students’ lived experience. J. Prev. Interv. Community.

[25] Mullins L., Preyde M. (2013). The lived experience of students with an invisible disability at a Canadian university. Disabil. Soc..

[26] Salzer M.S. (2012). A comparative study of campus experiences of college students with mental illnesses versus a general college sample. J. Am. Coll. Health.

[27] Appleby D.C., Appleby K.M. (2006). Kisses of death in the graduate school application process. Teach. Psychol..

[28] Harris J.I., Leskela J., Hoffman-Konn L. (2016). Provider lived experience and stigma. Am. J. Orthopsychiatry.

[29] Harris J.I., Barnes T., Boyd J.E., Joseph K., Osatuke K. (2022). Workplace bullying among mental health providers with lived experience of a mental health challenge. Psychol. Serv..

[30] King A.J., Brophy L.M., Fortune T.L., Byrne L. (2020). Factors affecting mental health professionals’ sharing of their lived experience in the workplace: A scoping review. Psychiatr. Serv..

[31] Haywood D., Baughman F., Mullan B., Heslop K.R. (2021). Going “Up” to Move Forward: S-1 Bifactor Models and the Study of Neurocognitive Abilities in Psychopathology. Int. J. Environ. Res. Public Health.

[32] Haywood D., Baughman F.D., Mullan B.A., Heslop K.R. (2021). Psychopathology and Neurocognition in the Era of the p-Factor: The Current Landscape and the Road Forward. Psychiatry Int..

[33] Haywood D., Baughman F.D., Mullan B.A., Heslop K.R. (2021). One p-Factor for All? Exploring the Applicability of Structural Models of Psychopathology within Subgroups of a Population. Int. J. Environ. Res. Public Health.

[34] Haywood D., Baughman F., Mullan B., Heslop K. (2022). What Accounts for the Factors of Psychopathology? An Investigation of the Neurocognitive Correlates of Internalising, Externalising, and the p-Factor. Brain Sci..

